# Cross-sectional analysis of self-reported sedentary behaviors and chronic knee pain among South Korean adults over 50 years of age in KNHANES 2013-2015

**DOI:** 10.1186/s12889-019-7653-9

**Published:** 2019-10-26

**Authors:** Sook-Hyun Lee, Chihyoung Son, Sujung Yeo, In-Hyuk Ha

**Affiliations:** 1 Jaseng Spine and Joint Research Institute, Jaseng Medical Foundation, Seoul, Republic of Korea; 2 Department of Oriental Rehabilitation Medicine, National Rehabilitation Center, Seoul, Republic of Korea; 3 Department of Meridian and Acupoint, College of Oriental Medicine, Sang Ji University, Wonju, Republic of Korea

**Keywords:** Chronic knee pain, Self-reported sedentary behavior, Physical activity, Body mass index, Korean National Health and nutrition examination survey

## Abstract

**Background:**

An increasing amount of evidence supports an association between sedentary behaviors and chronic knee pain. However, the association between the total daily duration of sedentary behavior and chronic knee pain in the general population remains unclear. We aimed to analyze the association between sedentary behavior and chronic knee pain in a study population representative of the general Korean population aged > 50 years while also considering the physical activity or body mass index (BMI).

**Methods:**

This cross-sectional study used data from the 6th Korean National Health and Nutrition Examination Survey (KNHANES VI) of 2013–2015, which was completed by 22,948 Korean adult participants aged > 50 years. The participants were divided into two groups based on the status of the chronic knee pain. Data were analyzed using multivariable logistic regression after adjustment for age, sex, and individual factors.

**Results:**

Longer sedentary behavior was correlated with chronic knee pain (p for trend = 0.02). Sedentary behavior exceeding 10 h/day was significantly associated with chronic knee pain (adjusted odds ratio, 1.28; *p* = 0.03). Participants with high levels of physical activity were less likely to suffer from chronic knee pain (adjusted odds ratio, 0.78; *p* = 0.00), and women with over 10 daily hours of sedentary behavior with high levels of physical activity were more likely to have chronic knee pain. A significant association was noted between chronic knee pain and obesity (≥30.0 kg/m^2^) individuals (adjusted odds ratio, 3.48; *p* = 0.04).

**Conclusions:**

Longer duration of sedentary behaviors was correlated with chronic knee pain. Our study suggests the need to encourage reductions in overall sedentary behavior to < 10 h daily. A high physical activity level is recommended, particularly for women > 50 years and those with obesity.

## Background

Osteoarthritis (OA) is the most common form of arthritis and the leading cause of disability globally, with pain being the main symptom. Most adults with OA experience associated pain. Particularly, the pain associated with knee OA develops from intermittent weight-bearing pain that evolves to chronic pain [1]. According to the clinical criteria of the American Rheumatology Society for the diagnosis of knee OA, age 50 years or older is considered a clinical diagnostic criterion [2]. Approximately 25% of the population aged 50 years or older experiences chronic pain from knee OA [3, 4]. The prevalence of OA was 12.5% ​​among adults > 50 years of age. The prevalence was 4.7% among patients in their 50s, 14.0% in those in their 60s, and 26.1% in those in their 70s [5].

OA imposes a substantial burden on individuals and society. The burden of OA and chronic knee pain includes direct costs (non-pharmacological or pharmacological treatment, surgery, adverse effects of treatment, long-term care), indirect costs (absenteeism, reduced employment, reduced productivity), and intangible costs (pain, activity limitations, decreased quality of life, fatigue, and reduced social participation) [6]. Chronic knee pain and disability owing to degenerative changes in the cartilage of older adults may reduce the quality of life and cause functional impairment, resulting in a loss of independence [7]. Given the general increase in life expectancy and prevalence of obesity, one would expect the prevalence of knee pain to increase concomitantly [8].

The etiology of chronic knee pain is multifactorial and complex, and several factors, including demographic, clinical, and psychological factors collectively contribute to its severity [9]. In the 2010 National Health and Nutrition Survey in Korea, the likelihood of chronic knee pain was associated with age, sex, low education, depression, obesity, and radiographic knee OA grade [10].

The decline in physical activity owing to the development of personal transportation (car, motorbike, etc.), increased accessibility to the internet, and increase in the prevalence of a sedentary lifestyle is gradually becoming a global problem [11]. The World Health Organization 2010 Physical activity-Health-Related Guidelines and the Physical Activity Guidelines for Americans 2018 also recommend replacing sedentary behavior with mild physical activity to prevent chronic disease and promote health [12, 13]. As reported in the 2015–2016 US National Health Nutrition Examination Survey, the average daily sitting time exceeds 8 h per day and inactivity increases with age [14]. It was also reported that 80% of the waking time is spent sitting [15, 16]; moreover, the elderly spent 5.3–9.4 h per waking day engaging in sedentary behaviors [17].

Sedentary behaviors are defined as activities that do not increase energy consumption much higher than those during resting (metabolic equivalents ≤1.5). These behaviors typically include activities, such as time spent sitting or lying down during waking hours [18, 19]. Specifically, in East Asia, including Korea, individuals commonly adopt seating postures on the floor, such as sitting cross-legged and kneeling [20]. The average adult spends about half of their waking time in a sitting position, perhaps because most occupations involve significant sitting time [21]. In addition, patients with chronic knee pain spend most of their waking hours engaged in sedentary behaviors, mostly because of pain and limitation of range of motion [22].

Physical activity is body movement resulting in energy expenditure by skeletal muscle contraction. Physical activity includes exercise and a wide range of sports as well as all body movements that occur during daily activities [13]. Based on the American Physical Therapy Association guidelines, moderate- to high-intensity exercises are recommended for patients with non-progressive knee pain, while low-intensity exercises are recommended for patients with generalized pain [13, 23]. Some studies reported that physical activity improves physical function and reduces pain and emphasized the importance of physical activity through guidelines.

The American College of Rheumatology Work Group Panel suggested that knee osteoarthritis patients engage in moderate-intensity (50–70% maximum heart rate) exercise 3 days per week [23]. Korea recommends low-impact aerobic exercise in its exercise guidelines [24].

According to the World Health Organization’s global recommendations on physical activity for health and Korea’s physical activity guidelines, adults aged 18–64 years and those over 65 years should complete at least 150 min of moderate-intensity aerobic physical activity per week, at least 75 min of vigorous-intensity aerobic physical activity per week, or an equivalent combination thereof [13, 25, 26]. Recent studies reported that more than half of Korean adults do meet the recommendation of at least 30 min of moderate activity on 3 or more days a week for at least 3 months [24].

Older people with chronic knee pain may have lower activity levels than the general population [27]. In the KNHANES V (2010–2012) of individuals aged ≥50 years with radiographic knee osteoarthritis, only 18.6% met the physical activity recommendations, a level that was significantly lower than the 23.3% reported for the general population [28]. Older people with chronic knee pain face difficulties performing physical activity and exercise owing to the chronic knee problem itself as well as social and individual factors [29]. To date, no study has investigated the relationship between sedentary behaviors and physical activity in the general Korean population.

Obesity can cause various complications and reportedly influences knee pain [30–32]. Previous studies on the relationship between obesity and duration of sedentary behaviors showed that obesity people tend to have a longer sitting time [33]. However, to the best of our knowledge no previous studies have reported the effects of obesity and sedentary behaviors on chronic knee pain.

This study aimed to analyze the association between the duration of sedentary behavior and chronic knee pain in a study population representative of the general Korean population aged over 50 years while also considering physical activity and obesity.

## Methods

### Participants

Data from Korea’s 6th National Health and Nutrition Examination Survey (KNHANES VI), 2013–2015 were used in this study. A nationwide, multistage, stratified, clustered, and random sampling method was used, which was proportionally distributed by sex, living area, and age of the Korean population.

**Fig. 1:**
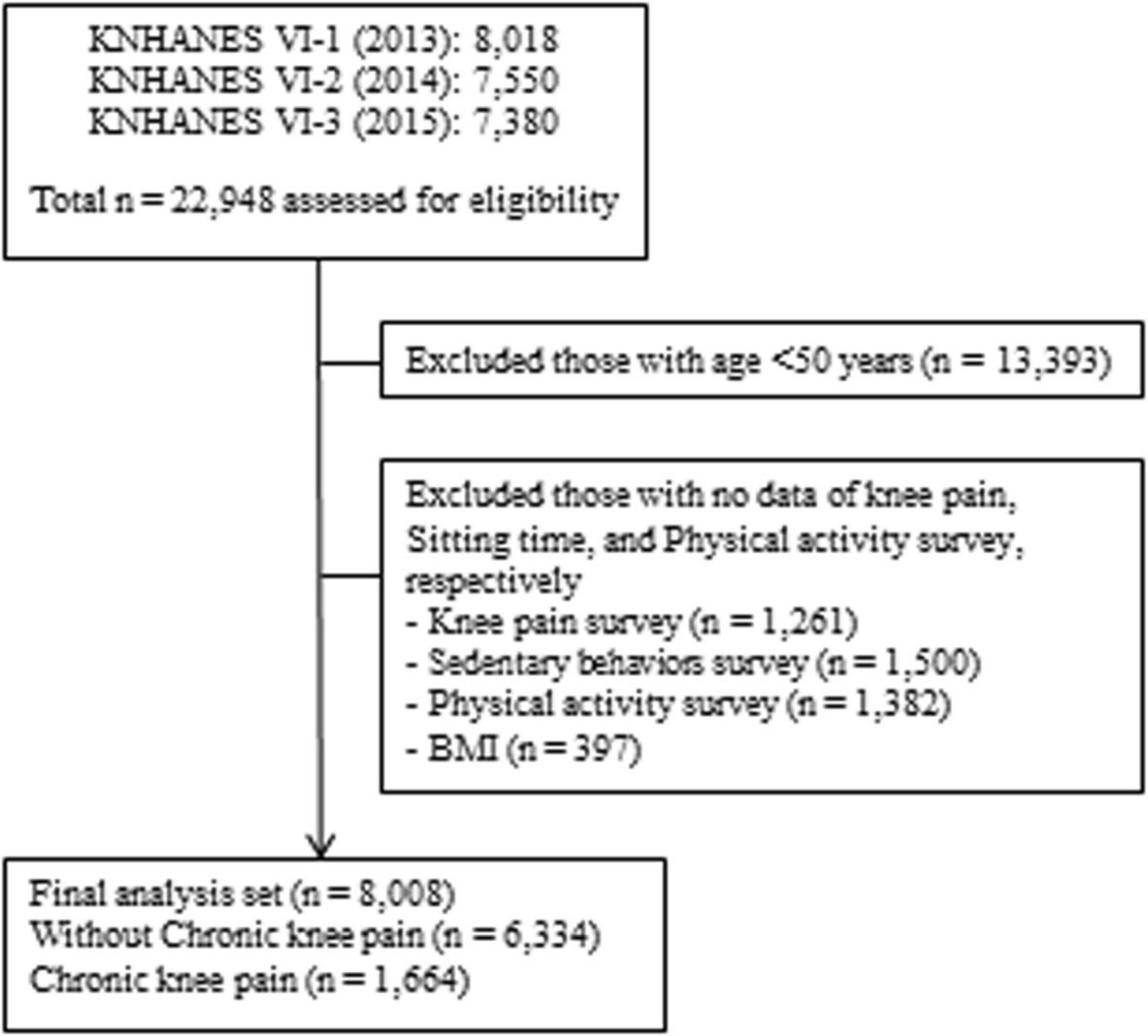
Flow diagram of the inclusion and exclusion criteria of participants from the 2013–2015 KNHANES VI-1–3

A total of 22,948 participants completed the KNHANES VI-1 (2013), VI-2 (2014), and VI-3 (2015) examinations and health surveys. The following participants were excluded: 1) those under 50 years of age and 2) those that did not respond to the chronic knee pain, sedentary behaviors, physical activity, and body mass index (BMI) surveys. Therefore, this analysis was confined to 8008 participants aged 50–89 years who answered the chronic knee pain examination survey and for whom no data regarding sedentary behaviors, physical activity, and BMI survey were missing (Fig. 1).

### Assessment of covariates

The participants’ demographic and socioeconomic characteristics and lifestyle were assessed using health interviews and examinations. Smoking status was categorized as no (non−/ex-smoker) or yes (current smoker) based on the present smoking status. Alcohol consumption was categorized as none, < 1 drink/month, 2 drinks/month to 3 drinks/week, or ≥ 4 drinks/month. Occupations were categorized as unemployed, office work, sales and services, agriculture, forestry and fishery, or machine fitting and simple labor. Household income was categorized into low, mid-low, mid-high, and high quartiles. Educational level was categorized as ≤6 years, 7–9 years, 10–12 years, and ≥ 13 years. Sleep duration was assessed using the following question: “How many hours per day do you usually sleep?”

### Chronic knee pain

Patients with chronic knee pain were evaluated using the following questions based on the classification criteria for idiopathic OA of the knee recommended by the American College of Rheumatology [23]: “Have you complained of knee joint pain for more than 30 days during the last 3 months?” Participants who answered “yes” were asked to describe the severity of the pain using a 0–10-point numerical rating scale [34].

### Sedentary behaviors

Sedentary behaviors were self-reported using the following question: How much time do you spend sitting or lying down during waking hours? The overall duration of sedentary behaviors was categorized as < 5, 5–7, 8–10, or > 10 h/day [35, 36].

### Physical activity

The International Physical Activity Questionnaire measures moderate- and vigorous-intensity physical activity and has tested reliability and validity with objective measurement tools [37]. High physical activity was defined as participating in moderate-intensity physical activity for at least 2 h and 30 min, vigorous-intensity physical activity for more than 1 h and 15 min, or a combination thereof (1 min of high-intensity activity is defined as 2 min of moderate-intensity activity) over a period of 1 week [25, 26]. A lesser level of physical activity was defined as low physical activity.

### BMI

BMI was categorized into underweight (< 18.5 kg/m^2^), normal weight (18.5–24.9 kg/m^2^), overweight (25.0–29.9 kg/m^2^), and obesity (≥30.0 kg/m^2^) [38].

### Statistical analysis

All statistical analyses were performed using SAS version 9.4 (SAS Institute Inc., Cary, NC, USA) and consisted of complex sample analysis–stratified cluster sampling with weighted samples.

General characteristics were compared between participants with and without chronic knee pain using Student’s t-test and Chi-square test. The latter was used to examine the likelihood associated with chronic knee pain. Furthermore, to identify factors associated with chronic knee pain, odds ratios (ORs) and 95% confidence intervals (CIs) were calculated through multivariable logistic regression analysis after setting chronic knee pain as the dependent variable and sedentary behaviors as the main independent variable with control for sociodemographic, lifestyle, and health factors. The association between sedentary behavior and chronic knee pain was analyzed using subgroups based on sex, physical activity and BMI. The *p* values of all analyses performed in this study were two-tailed with a significance level of < 0.05.

## Results

### Participant demographics according to chronic knee pain status

The characteristics of the study population from the survey with and without chronic knee pain are shown in Table 1. A total of 8008 participants were finally analyzed, including 1664 with chronic knee pain and 6344 without. The prevalence of chronic knee pain was 20.8% among Korean participants older than 50 years. The majority of the participants were women; of whom, 27.4% had chronic knee pain. In contrast, only 12% of the men patients had chronic knee pain. The participants with chronic knee pain were older (65.2 ± 9.3 years) than those without (61.3 ± 8.7 years) (*p* < 0.0001). The low PA group (22.6%) had a higher prevalence of chronic knee pain than the high PA group (17.2%) (*p* < 0.0001). Participants with chronic knee pain demonstrated longer hours of daily sedentary behavior (6.9 ± 3.8 h) than those without chronic knee pain (6.5 ± 3.6 h) (*p* < 0.0001).

**Table 1:** Characteristics of the study population from the survey with and without chronic knee pain

Variable	Without chronic knee pain n (%)	Chronic knee pain n (%)	*P*-value
Total	6344 (79.2)	1664 (20.8)	
Age (y)	61.3 ± 8.7	65.2 ± 9.3	<.0001
Age, *n* (%)			
50–59	2583 (85.8)	428 (14.2)	<.0001
60–69	2098 (79.4)	543 (20.6)	
70–79	1373 (71.8)	539 (28.2)	
≥ 80	290 (65.3)	154 (34.7)	
Sex, *n* (%)			
Men	3024 (88)	411 (12)	<.0001
Women	3320 (72.6)	1253 (27.4)	
BMI (kg/m^2^)	24.0 ± 3.1	24.7 ± 3.3	<.0001
BMI (categorical), *n* (%)^a^			
Underweight (< 18.5)	171 (83.0)	35 (17.0)	<.0001
Normal (18.5–24.9)	3984 (81.2)	922 (18.8)	
Overweight (25.0–29.9)	1968 (76.6)	601 (23.4)	
Obesity (≥30)	221 (67.6)	106 (32.4)	
Smoking status, *n* (%)			
No	5308 (78.4)	1464 (21.6)	<.0001
Yes	965 (85.1)	169 (14.9)	
Alcohol consumption, *n* (%)			
None	2333 (74.8)	787 (25.2)	<.0001
≤ 1 drink/mo	1037 (77.9)	295 (22.2)	
2 drinks/mo to 3 drinks/wk	1576 (83.2)	319 (16.8)	
≥ 4 drinks/wk	1334 (85)	236 (15)	
Occupation, *n* (%)			
Unemployed (student, housewife, etc.)	2974 (74.7)	1006 (25.3)	<.0001
Office work	701 (92.1)	60 (7.9)	
Sales and services	720 (85.2)	125 (14.8)	
Agriculture, forestry, and fishery	505 (75.3)	166 (24.7)	
Machine fitting and simple labor	1430 (82.6)	302 (17.4)	
Household income, *n* (%) ^b^			
Low	1608 (69.1)	719 (30.9)	<.0001
Mid-low	1702 (79.8)	431 (20.2)	
Mid-high	1441 (83.3)	289 (16.7)	
High	1566 (87.9)	216 (12.1)	
Education, *n* (%)			
≤ 6 y	2326 (68.8)	1057 (31.2)	<.0001
7–9 y	1127 (80.9)	266 (19.1)	
10–12 y	1792 (87.5)	256 (12.5)	
≥ 13 y	1086 (93.2)	79 (6.8)	
Duration of sleep (h), mean ± sd	6.6 ± 1.4	6.4 ± 1.7	<.0001
Depressive symptom, *n* (%) ^c^	307 (64.6)	168 (35.4)	<.0001
Physical activity, n(%)			
Low	4083 (77.4)	1195 (22.6)	<.0001
High	2261 (82.8)	469 (17.2)	
Sedentary behaviors (h), mean ± sd	6.5 ± 3.6	6.9 ± 3.8	0.0145
Sedentary behaviors (h) ^d^			
< 5	2123 (33.5)	492 (29.6)	0.0909
5–7	1950 (30.7)	498 (29.9)	
8–10	1474 (23.2)	415 (24.9)	
> 10	797 (12.6)	259 (15.6)	

*BMI* body mass index

Numeric parameters are expressed as means with standard deviation in parentheses, unless stated otherwise. Categorical parameters are expressed as counts with percentages in parentheses

^a^ BMI was categorized into underweight (< 18.5 kg/m^2^), normal (18.5–24.9 kg/m^2^), overweight (25.0–29.9 kg/m^2^), and obesity (≥30.0 kg/m^2^)

^b^ Household income levels were calculated by dividing the total household monthly income with the obtained levels then grouped into quartiles

^c^ Depressive symptoms were defined as individuals who felt sad or experienced other depressive symptoms for 2 consecutive weeks during the past year

^d^ Levels of sedentary behaviors were categorized using quartiles: < 5, 5–7, 8–10, and > 10 h/day

The mean age of the BMI in the group with chronic knee pain (24.7 ± 3.3 kg/m^2^) were older than those in the group without chronic knee pain (24.0 ± 3.1 kg/m^2^) (*p* < 0.0001). In the chronic knee pain group, the prevalence of obesity (30 kg/m^2^) was the highest at 32.4%, followed by overweight (25.0–29.9 kg/m^2^) (*p* < 0.0001).

Overall, 29.6, 29.9, 24.9, and 15.6% of the participants with chronic knee pain reported sedentary behaviors for < 5, 5–7, 8–10, and > 10 h/day, respectively. Age, sex, BMI, symptoms of depression, current smoking status, alcohol consumption, occupation, household income, education level, physical activity, and sleep duration were significantly different between participants with and those without chronic knee pain among Korean participants aged > 50 years. Participants with chronic knee pain were more likely to be older, women, non-smokers, and unemployed with lower income and lower education levels. In terms of lifestyle, participants with chronic knee pain demonstrated lower levels of PA than those without chronic knee pain.

### ORs of sedentary behaviors for chronic knee pain by sex

Table 2 shows the association between sedentary behaviors and chronic knee pain using multivariable logistic regression by sex. Model 1 was adjusted for age and BMI. Model 2 was adjusted for individual factors (lifestyle factors and health factors), such as smoking, alcohol consumption, occupation, education, household income, physical activity, depression, and sleep duration as well as the factors of Model 1.

**Table 2:** Association between sedentary behaviors and chronic knee pain using multivariable logistic regression by sex

Sedentary behaviors 4 categories ^a^	Unadjusted		Model 1		Model 2	
	OR (95% CI)	*P*-value	OR (95% CI)	*P*-value	OR (95% CI)	*P*-value
Total						
< 5	1		1		1	
5–7	1.02 (0.86–1.20)	0.78	0.99 (0.84–1.159)	0.87	1.03 (0.87–1.22)	0.73
8–10	1.11 (0.93–1.33)	0.24	1.05 (0.87–1.26)	0.64	1.17 (0.96–1.42)	0.12
> 10	1.28 (1.03–1.60)	0.03	1.21 (0.97–1.51)	0.1	1.28 (1.02–1.61)	0.03
p for trend	1.08 (1.01–1.15)	0.02	1.05 (0.99–1.13)	0.12	1.09 (1.02–1.16)	0.02
Men						
< 5	1		1		1	
5–7	0.83 (0.62–1.12)	0.22	0.81 (0.60–1.08)	0.15	0.89 (0.63–1.25)	0.50
8–10	0.93 (0.67–1.29)	0.66	0.92 (0.66–1.28)	0.61	1.11 (0.78–1.58)	0.57
> 10	1.02 (0.67–1.56)	0.93	0.99 (0.64–1.52)	0.96	1.17 (0.78–1.75	0.46
p for trend	1.00 (0.88–1.14)	0.98	0.99 (0.87–1.13)	0.92	1.06 (0.94–1.20)	0.36
Women						
< 5	1		1		1	
5–7	1.11 (0.91–1.35)	0.31	1.06 (0.86–1.30)	0.60	1.12 (0.91–1.38)	0.30
8–10	1.21 (0.98–1.50)	0.08	1.08 (0.87–1.35)	0.48	1.21 (0.96–1.52)	0.10
> 10	1.56 (1.22–2.00)	0.00	1.27 (0.99–1.63)	0.07	1.33 (1.02–1.74)	0.04
p for trend	1.14 (1.06–1.23)	0.00	1.07 (0.99–1.15)	0.10	1.10 (1.01–1.19)	0.02

Multivariable Logistic regression analysis with complex sampling design was performed by adjusting for covariates. OR, odds ratio; 95% CI, 95% confidence interval

^a^ Levels of sedentary behaviors were categorized using quartiles: < 5, 5–7, 8–10, and > 10 h/day

Model 1 was adjusted for age and BMI

Model 2 was adjusted for Model 1 + smoking, alcohol consumption, occupation, education, household income, physical activity, depression, and duration of sleep

Overall, the ORs of chronic knee pain for participants with > 10 h/day of sedentary behaviors was 1.28 (95% CI, 1.03–1.60) for unadjusted and 1.28 (95% CI, 1.02–1.61) for Model 2, showing a significant association between chronic knee pain and sedentary behavior of > 10 h/day (*p* = 0.03 and *p* = 0.03, respectively). Among women participants, a significant association between chronic knee pain and > 10 h/day of sedentary behavior was seen (*p* = 0.00 and *p* = 0.04, respectively). The ORs of chronic knee pain for > 10 h/day of sedentary behaviors were 1.56 (95% CI, 1.22–2.00) for unadjusted model and 1.33 (95% CI, 1.02–1.74) for Model 2. Regarding the *p*-value for trend, the ORs for chronic knee pain increased with increase in the level of sedentary behavior in unadjusted model (*p* = 0.00) and in Model 2 (*p* = 0.02). Among men, there was no significant association between chronic knee pain and level of sedentary behavior.

### ORs of sedentary behaviors for chronic knee pain according to physical activity by sex

Table 3 shows the association between physical activity and chronic knee pain using multivariable logistic regression by sex. Model 1 was adjusted for age and BMI, while Model 2 was adjusted for individual factors (lifestyle factors and health factors), such as smoking, alcohol consumption, education, household income, depression, and sleep duration as well as the factors of Model 1.

**Table 3:** Association between physical activity and chronic knee pain using multivariable logistic regression by sex

Physical activity 2 categories^a^	Unadjusted		Model 1		Model 2	
	OR (95% CI)	*P*-value	OR (95% CI)	*P*-value	OR (95% CI)	*P*-value
Total						
Low physical activity	1		1		1	
High physical activity	0.64 (0.55–0.73)	0.00	0.78 (0.67–0.91)	0.00	0.86 (0.74–1.01)	0.06
Men						
Low physical activity	1		1		1	
High physical activity	0.68 (0.52–0.89)	0.01	0.70 (0.54–0.92)	0.01	0.79 (0.60–1.04)	0.10
Women						
Low physical activity	1		1		1	
High physical activity	0.69 (0.59–0.82)	0.00	0.84 (0.70–1.00)	0.04	0.90 (0.75–1.08)	0.27

Multivariable Logistic regression analysis with complex sampling design was performed by adjusting for covariates. OR, odds ratio; 95% CI, 95% confidence interval

^a^ Levels of physical activity were categorized: high physical activity was defined as participating in moderate-intensity physical activity for at least 2 h 30 min, vigorous-intensity physical activity for more than 1 h 15 min, or a combination of moderate and high-intensity physical activity (1 min of high-intensity activity is defined as 2 min of moderate-intensity activity) over a period of 1 week. Physical activity less than that mentioned previously was defined as low physical activity

Model 1 was adjusted for age and BMI

Model 2 was adjusted for Model 1 + smoking, alcohol consumption, education, household income, depression, and duration of sleep

Overall, the ORs of chronic knee pain for high physical activity were 0.64 (95% CI, 0.55–0.73) for unadjusted model and 0.78 (95% CI, 0.67–0.91) for Model 1. In the total population, a significant association was noted between chronic knee pain and high physical activity (*p* = 0.00 and *p* = 0.00, respectively). A significant association was noted between chronic knee pain and high physical activity (*p* = 0.01 and *p* = 0.01, respectively) among men participants. The ORs of chronic knee pain for high physical activity were 0.68 (95% CI, 0.52–0.89) for unadjusted model and 0.70 (95% CI, 0.54–0.92) for Model 1. Women participants showed a significant association between chronic knee pain and physical activity (*p* = 0.00 and *p* = 0.04, respectively). The ORs of chronic knee pain for high physical activity were 0.69 (95% CI, 0.59–0.82) for unadjusted model and 0.84 (95% CI, 0.70–1.00) for Model 1. However, the associations for total, men, and women participants in the full adjusted model 2 were not significant.

### ORs of sedentary behaviors for chronic knee pain by BMI

Table 4 shows the association between BMI and chronic knee pain using multivariable logistic regression by sex. Model 1 was adjusted for age and model 2 was adjusted for individual factors (lifestyle factors and health factors), such as smoking, alcohol consumption, education, household income, physical activity, depression, and sleep duration as well as factors of Model 1.

**Table 4:** Association between BMI and chronic knee pain using multivariable logistic regression by sex

BMI 4 categories^a^	Unadjusted		Model 1		Model 2	
	OR (95% CI)	*P*-value	OR (95% CI)	*P*-value	OR (95% CI)	*P*-value
Total						
Underweight	1		1		1	
Normal	1.18 (0.77–1.83)	0.45	1.38 (0.89–2.16)	0.15	1.53 (0.95–2.46)	0.08
Overweight	1.48 (0.95–2.32)	0.09	1.79 (1.13–2.84)	0.01	1.86 (1.14–3.04)	0.01
Obesity	2.44 (1.48–4.01)	0.00	2.64 (1.59–4.38)	0.00	2.45 (1.43–4.21)	0.00
P for trend	1.32 (1.20–1.46)	0.00	1.34 (1.21–1.48)	0.00	1.26 (1.14–1.40)	0.00
Men						
Underweight	1		1		1	
Normal	1.83 (0.74–4.54)	0.19	0.70 (0.54–0.92)	0.01	0.79 (0.60–1.04)	0.10
Overweight	1.58 (0.63–3.96)	0.33	1.96 (0.77–4.94)	0.16	2.32 (0.83–6.51)	0.11
Obesity	2.44 (0.82–7.28)	0.11	3.17 (1.06–9.42)	0.04	3.48 (1.03–11.74)	0.04
P for trend	1.00 (0.81–1.23)	0.99	1.07 (0.87–1.31)	0.54	1.07 (0.87–1.32)	0.50
Women						
Underweight	1		1		1	
Normal	0.97 (0.61–1.54)	0.90	1.13 (0.71–1.81)	0.61	1.17 (0.71–1.93)	0.53
Overweight	1.53 (0.94–2.48)	0.09	1.71 (1.04–2.79)	0.03	1.62 (0.97–2.71)	0.07
Obesity	1.97 (1.12–3.45)	0.02	2.36 (1.35–4.14)	0.00	2.01 (1.12–3.62)	0.02
P for trend	1.45 (1.29–1.63)	0.00	1.45 (1.28–1.63)	0.00	1.33 (1.18–1.51)	0.00

Multivariable Logistic regression analysis with complex sampling design was performed by adjusting for covariates. OR, odds ratio; 95% CI, 95% confidence interval

^a^ BMI was categorized into underweight (< 18.5 kg/m^2^), normal weight (18.5–24.9 kg/m^2^), overweight (25.0–29.9 kg/m^2^), and obesity (≥30.0 kg/m^2^)

Model 1 was adjusted for age

Model 2 was adjusted for Model 1 + smoking, alcohol consumption, occupation, education, household income, physical activity, depression, and duration of sleep

Overall, the ORs of chronic knee pain and overweight (25.0–29.9 kg/m^2^) were 1.79 (95% CI, 1.13–2.84) and 1.86 (95% CI, 1.14–3.04) for Model 1 and Model 2, respectively. A significant association between chronic knee pain and overweight (25.0–29.9 kg/m^2^) (*p* = 0.01 and *p* = 0.01, respectively) was noted. The ORs of chronic knee pain and obesity (≥30.0 kg/m^2^) were 2.44 (95% CI, 1.48–4.01) for unadjusted model, 2.64 (95% CI, 1.59–4.38) for model 1, and 2.45 (1.43–4.21) for Model 2. A significant association was noted between chronic knee pain and obesity (≥30.0 kg/m^2^) (*p* = 0.00, *p* = 0.00, and *p* = 0.00, respectively). In p for trend, the chronic knee pain correlated with BMI in unadjusted model (OR, 1.32; 95% CI, 1.20–1.46; *p* = 0.00), Model 1 (OR, 1.34; 95% CI, 1.21–1.48; *p* = 0.00) and in Model 2 (OR, 1.34; 95% CI, 1.14–1.40; *p* = 0.00).

Among men participants, a significant association was noted between chronic knee pain and obesity (≥30.0 kg/m^2^) (*p* = 0.04). The ORs of chronic knee pain for obesity (≥30.0 kg/m^2^) were 3.17 (95% CI, 1.06–9.42) for Model 1 and 3.48 (95% CI, 1.03–11.74) for Model 2. Among the women participants, a significant association was noted between chronic knee pain and obesity (≥30.0 kg/m^2^). The ORs of chronic knee pain for obesity were 1.97 (95% CI, 1.12–3.45) for unadjusted model, 2.36 (95% CI, 1.35–4.14) for Model 1, and 2.01 (95% CI, 1.12–3.62) for Model 2. Further, the women participants showed a significant association between chronic knee pain and BMI (*p* = 0.02, 0.00, and 0.02). In p for trend, chronic knee pain was correlated to BMI in unadjusted model (OR, 1.45; 95% CI, 1.29–1.63; *p* = 0.00), Model 1 (OR, 1.45; 95% CI, 1.28–1.63; *p* = 0.00) and Model 2 (OR, 1.33; 95% CI, 1.18–1.51; *p* = 0.00).

### ORs of sedentary behaviors and chronic knee pain by high physical activity using multivariable logistic regression by sex

Additional file 1: Table S1 shows the association between chronic knee pain and sedentary behaviors based on the high levels of physical activity multivariable logistic regression by sex. Model 1 was adjusted for age and individual factors (lifestyle factors and health factors), such as BMI, smoking, alcohol consumption, occupation, education, household income, depression, and duration of sleep.

In the subgroup analysis, the association between chronic knee pain and sedentary behaviors based on high physical activity and sex was analyzed in more detail using multivariable logistic regression (Additional file 1: Table S1). Women participants showed a significant association between chronic knee pain and high physical activity (*p* = 0.04). The OR of chronic knee pain for high levels of physical activity was 1.33 (95% CI, 1.02–1.74) in Model 3. In p for trend, chronic knee pain correlated with sedentary behaviors at high physical activity in unadjusted model (OR, 1.15; 95% CI, 1.00–1.32; *p* = 0.04) and Model 1 (OR, 1.19; 95% CI, 1.02–1.39; *p* = 0.03).

### ORs ratios of sedentary behaviors and chronic knee pain using multivariable logistic regression by sex

Additional file 2: Table S2 shows the association between chronic knee pain and sedentary behaviors according to BMI using multivariable logistic regression by sex. Model 1 was adjusted for age and individual factors (lifestyle factors and health factors), such as smoking, alcohol consumption, occupation, education, household income, physical activity, depression, and duration of sleep.

In subgroup analysis, the association between chronic knee pain and sedentary behaviors by BMI and sex was analyzed in further detail using multivariable logistic regression (Additional file 2: Table S2). Women participants showed significant association between chronic knee pain and BMI (*p* = 0.04). The ORs of chronic knee pain for high physical activity was 1.37 (95% CI, 1.02–1.84) in Model 1. Meanwhile, women showed no statistically significant differences between sedentary behavior and chronic pain when overweight or obesity. Men participants also showed no statistically significant differences between sedentary behavior and chronic pain with normal weight or overweight. In obesity men participants, statistical analysis could not be performed owing to lack of numbers.

## Discussion

In this study, we analyzed the relationship between sedentary behavior and chronic knee pain among 8008 participants using reliable data that were representative of the general Korean population. Longer sedentary behavior and BMI were correlated with chronic knee pain.

In addition, even with high levels of physical activity, longer sedentary behavior (over than 10 h/day) was associated with chronic knee pain among women.

A previous study that analyzed data from the KNHANES in 2010–2012 reported that age, women sex, lower education level, obesity, and depression were risk factors for knee pain in adults > 50 years of age [39]. This is consistent with our findings. In our study, participants with chronic knee pain were older, women, non-smokers, and unemployed with lower income and education levels. Further, participants with chronic knee pain had lower levels of physical activity than those without chronic knee pain.

Knee joints can become stiff while getting out of bed in the morning or after long periods of sitting [40]. OA pain in the knee can lead to knee instability (buckling) owing to secondary patellar imbalance caused by weakening of the quadriceps muscle [41, 42]. In addition, the decrease in quadriceps muscle strength can cause difficulty in movements, such as standing from the sitting position. Continuation of this condition may cause flexion deformation in the knee joint [43]. Knee stiffness limits extension movements, causes an increase in the stress applied to the joints, induces secondary pain, reduces muscle flexibility, and aggravates knee OA by creating a pain loop [44]. Thus, the individual may experience difficulty while performing everyday life activities, such as climbing stairs and self-care.

An earlier clinical study examined the correlation between longer sedentary behaviors and arthritis among obesity men participants and found that longer periods of sedentary behaviors gradually resulted in weakness of the quadriceps muscle, resulting in wear and tear in the area below the patella, causing pain [40]. In one study, obesity and chronic pain were frequently associated with each other, negatively affected each other, and were mediated by a variety of factors (i.e. lifestyle factors, including inflammatory intermediates, mood disorders, sleep deprivation, and biomechanical structural changes). The findings of our study also showed that individual factors (lifestyle and health factors) such as smoking, alcohol consumption, education, household income, physical activity, depression, and sleep duration affected knee pain. Furthermore, our study confirmed the relationship between obesity and knee pain and examined the relationship between sedentary behavior and obesity in subjects with knee pain. Consistent with these findings, our study suggested that obesity individuals were more likely to suffer from chronic knee pain. However, women with normal weight were 1.37 times more likely to suffer from chronic knee pain with 8–10 h of sedentary behavior than those with < 5 h of sedentary behaviors.

Promotion of health among the elderly by emphasizing on moderate-intensity aerobic and muscle strengthening activities and by reducing sedentary behaviors is encouraged based on the recommendations of the American College of Sports Medicine and American Heart Association [25]. This is consistent with our results that emphasize the need to reduce sedentary behaviors among adults > 50 years of age. In addition, according to previous reports on exercise and physical activity behaviors, 44% of individuals with knee pain reported low levels of physical activity. Our study showed that individuals in the high physical activity group showed lesser tendency for knee pain [27]. In other words, there was a high incidence of chronic knee pain in the low physical activity group, a finding consistent with that in previous studies.

This study had a number of advantages. First, it was based on the reliable data obtained from the Korean government agencies from questionnaires completed by a sample representative of the general Korean population. Second, this was the first study to investigate the relationship between sedentary behaviors and chronic knee pain based on levels of physical activity or BMI. Third, individuals in East Asia, including Korea, commonly sit on the floor in a crossed-leg or kneeling posture; therefore, research investigating the effect of sedentary behaviors on knee pain is important.

There were some limitations to our research. First, this study used a cross-sectional design to analyze a sample of the general population. Therefore, a causal relationship could not be explored. However, this study aimed to observe the general population in Korea. Sampling error was minimized using a clustering multistage random sampling method. Second, the brief questionnaire used in this study to evaluate chronic knee pain did not describe its severity, source, or duration, probably by using a pain measurement tool with a scale (e.g. a numerical rating scale). Third, the relationship between sedentary behaviors and chronic knee pain determined here was dependent on ethnicity. The KNHANES is a nationwide survey targeting the general public in Korea. Therefore, as our findings are representative of the general Korean population, extrapolating our results to other populations may not be valid. Fourth, the confounders (age, BMI, smoking, alcohol consumption, occupation, education, household income, physical activity, depression, and sleep duration) used in this study did not significantly influence chronic knee pain; thus, they were used only as control variables. We did not identify the multicollinearity statistically because the confounders used in this study were considered to have little influence on chronic knee pain [1, 10, 45]. Future research must include statistical analysis of multicollinearity. Finally, the data obtained in this study from the KNHANES VI-1, VI-2, and VI-3 surveys were limited as the chronic knee pain survey was completed only by patients aged > 50 years.

## Conclusions

In conclusion, sedentary behaviors are significantly positively correlated with chronic knee pain. Obesity individuals with low physical activity and longer sedentary behaviors are more likely to suffer from chronic knee pain. However, among women participants who demonstrated high levels of physical activity, being sedentary for more than 10 h was more likely to cause chronic knee pain. Thus, the findings of our population-based study suggest that reductions in overall sedentary behaviors should be encouraged, particularly among individuals with chronic knee pain. High levels of physical activity are, particularly for women > 50 years old and those with obesity.

## Supplementary information

**Additional file 1: Table S1.** Association between sedentary behaviors and chronic knee pain according to high physical activity using multivariable logistic regression by sex.

**Additional file 2: Table S2.** Association between sedentary behaviors and chronic knee pain according to BMI using multiple logistic regression by sex.

## Data Availability

Survey data are publicly available at (https://knhanes.cdc.go.kr/knhanes/main.do). All data from KNHANES VI are coded and freely available.

## Abbreviations

BMI: Body mass index
CIs: Confidence intervals
KNHANES: Korea National Health and Nutrition Examination Survey
ORs: Odds ratios
