# Supplementary material for: Cross-sectional analysis of self-reported sedentary behaviors and chronic knee pain among South Korean adults over 50 years of age in KNHANES 2013-2015
Source: BMC Public Health. 2019 Oct 26;19:1375. doi: 10.1186/s12889-019-7653-9 (PMC6815384; doi:10.1186/s12889-019-7653-9)
Supplement: Supplementary file 1 — Additional file 1: Table S1. Association between sedentary behaviors and chronic knee pain according to high physical activity using multivariable logistic regression by sex. [file 12889_2019_7653_MOESM1_ESM.docx]

Supplementary Table 1. Association between sedentary behaviors and chronic knee pain according to high physical activity using multivariable logistic regression by sex

| Sedentary behaviors  4 categories ^1)^ | Unadjusted | | Model 1 | |
| --- | --- | --- | --- | --- |
|  | OR (95% CI) | *P*-value | OR (95% CI) | *P*-value |
| **High physical activity** ^2)^ |  |  |  |  |
| **Men** | | | | |
| <5 | 1 | | 1 | |
| 5–7 | 0.98 (0.59 - 1.61) | 0.86 | 1.07 (0.61 - 1.86) | 0.82 |
| 8–10 | 0.81 (0.45 - 1.44) | 0.42 | 1.06 (0.55 - 2.05) | 0.86 |
| >10 | 1.01 (0.48 - 2.14) | 0.79 | 1.34 (0.65 - 2.79) | 0.43 |
| p for trend | 0.97 (0.77 - 1.21) | 0.76 | 1.08 (0.85 - 0.37) | 0.53 |
| **Women** | | | | |
| <5 | 1 | | 1 | |
| 5–7 | 1.29 (0.89 - 1.89) | 0.94 | 1.11 (0.90 - 1.37) | 0.31 |
| 8–10 | 1.33 (0.89 - 2.00) | 0.76 | 1.21 (0.96 - 1.52) | 0.10 |
| >10 | 1.57 (0.99 - 2.50) | 0.20 | **1.33 (1.02** - **1.74)** | **0.04** |
| p for trend | **1.15 (1.00** - **1.32)** | **0.04** | **1.19 (1.02** - **1.39)** | **0.03** |

Multivariable Logistic regression analysis with complex sampling design was performed by adjusting for covariates. OR, odds ratio; 95% CI, 95% confidence interval.

Values are presented as OR(95% CI); OR(95% CI) by multivariable logistic regression analyses.

^1)^ Levels of sedentary behaviors were categorized using quartiles: <5, 5–7, 8–10, and >10 hours/day.

^2)^ Levels of physical activity (PA) were categorized: High PA was defined as participating in moderate-intensity PA for at least 2 hours 30 minutes, vigorous-intensity PA for more than 1 hour 15 minutes, or a combination of moderate and high-intensity PA (1 minute of high-intensity activity is defined as 2 minutes of moderate-intensity activity) over a period of one week.

Model 1 was adjusted for age, BMI, smoking, alcohol consumption, education, household income, depression, and duration of sleep.
