# Supplementary material for: Cross-sectional analysis of self-reported sedentary behaviors and chronic knee pain among South Korean adults over 50 years of age in KNHANES 2013-2015
Source: BMC Public Health. 2019 Oct 26;19:1375. doi: 10.1186/s12889-019-7653-9 (PMC6815384; doi:10.1186/s12889-019-7653-9)
Supplement: Supplementary file 2 — Additional file 2: Table S2. Association between sedentary behaviors and chronic knee pain according to BMI using multiple logistic regression by sex. [file 12889_2019_7653_MOESM2_ESM.docx]

Supplementary Table 2. Association between sedentary behaviors and chronic knee pain according to BMI using multiple logistic regression by sex

| Sedentary behaviors  4 categories ^1)^ | Unadjusted | | Model 1 | |
| --- | --- | --- | --- | --- |
|  | OR (95% CI) | *P*-value | OR (95% CI) | *P*-value |
| **BMI** ^2)^ |  |  |  |  |
| **Men** | | | | |
| **Normal** | | | | |
| <5 | 1 | | 1 | |
| 5–7 | 0.84 (0.58 - 1.21) | 0.59 | 0.85 (0.56 - 1.29) | 0.44 |
| 8–10 | 0.75 (0.49 - 1.14) | 0.21 | 0.87 (0.55 - 1.38) | 0.55 |
| >10 | 1.05 (0.61 - 1.82) | 0.43 | 1.07 (0.64 - 1.81) | 0.79 |
| p for trend | 0.97 (0.82 – 1.15) | 0.73 | 1.00 (0.84 - 1.18) | 0.99 |
| **Overweight** | | | | |
| <5 | 1 | | 1 | |
| 5–7 | 0.66 (0.39 - 1.12) | 0.09 | 0.82 (0.47 - 1.45) | 0.49 |
| 8–10 | 1.24 (0.72 - 2.13) | 0.09 | 1.69 (0.95 - 3.00) | 0.08 |
| >10 | 0.80 (0.39 - 1.64) | 0.64 | 1.26 (0.59 - 2.67) | 0.55 |
| p for trend | 1.00 (0.81 - 1.24) | 0.98 | 1.17 (0.94 – 1.45) | 0.15 |
| **Women** | | | | |
| **Normal** | | | | |
| <5 | 1 | | 1 | |
| 5–7 | 1.18 (0.91 - 1.53) | 0.49 | 1.21 (0.92 - 1.59) | 0.18 |
| 8–10 | 1.34 (1.02 - 1.78) | 0.43 | **1.37 (1.02 - 1.84)** | **0.04** |
| >10 | 1.55 (1.12 - 2.14) | 0.06 | 1.26 (0.90 - 1.77) | 0.18 |
| p for trend | **1.16 (1.05 - 1.28)** | **0.00** | 1.11 (1.00 - 1.23) | 0.05 |
| **Overweight** | | | | |
| <5 | 1 | | 1 | |
| 5–7 | 1.00 (0.71- 1.41) | 0.33 | 1.06 (0.74 - 1.51) | 0.76 |
| 8–10 | 1.07 (0.75 - 1.55) | 0.76 | 1.09 (0.73 - 1.62) | 0.67 |
| >10 | 1.43 (0.94- 2.18) | 0.08 | 1.38 (0.87 - 2.20) | 0.17 |
| p for trend | 1.10 (0.97 - 1.25) | 0.14 | 1.09 (0.95 – 1.26) | 0.23 |
| **Obesity** | | | | |
| <5 | 1 | | 1 | |
| 5–7 | 0.91 (0.42- 1.95) | 0.46 | 0.62 (0.26 - 1.49) | 0.29 |
| 8–10 | 0.88 (0.39 - 1.97) | 0.42 | 0.70 (0.32 - 1.55) | 0.38 |
| >10 | 1.78 (0.73 - 4.34) | 0.11 | 1.40 (0.50 – 3.92) | 0.53 |
| p for trend | 1.15 (0.87 - 1.53) | 0.32 | 1.07 (0.79 – 1.44) | 0.68 |

Multivariable Logistic regression analysis with complex sampling design was performed by adjusting for covariates. OR, odds ratio; 95% CI, 95% confidence interval.

^1)^ Levels of sedentary behaviors were categorized using quartiles: <5, 5–7, 8–10, and >10 hours/day.

^2)^ BMI was categorized into normal weight (18.5–24.9 kg/m^2^), and overweight (25.0–29.9 kg/m^2^), obesity (≥30.0 kg/m^2^).

Model 1 was adjusted by age, smoking, alcohol consumption, occupation, education, household income, physical activity, depression, and duration of sleep.
